# Pre-pandemic care-seeking patterns and subsequent diagnoses of post-COVID condition, post viral fatigue syndrome, and exhaustion disorder: a registry-based cohort study of 208,050 Swedish women

**DOI:** 10.1080/02813432.2025.2611886

**Published:** 2026-01-06

**Authors:** Agnes af Geijerstam, Kirsten Mehlig, Fredrik Nyberg, Annika Rosengren, Ailiana Santosa, Maria Åberg, Lauren Lissner

**Affiliations:** ^a^ School of Public Health and Community Medicine, Institute of Medicine, Sahlgrenska Academy, University of Gothenburg, Gothenburg, Sweden; ^b^ Department of Molecular and Clinical Medicine, Institute of Medicine, Sahlgrenska Academy, University of Gothenburg, Gothenburg, Sweden; ^c^ Department of Medicine, Geriatrics and Emergency Medicine, Region Västra Götaland, Sahlgrenska University Hospital/Östra, Gothenburg, Sweden; ^d^ Region Västra Götaland, Regionhälsan, Gothenburg, Sweden

**Keywords:** Post-COVID condition, post-viral fatigue syndrome, exhaustion disorder, primary-care utilization, care-seeking behaviour, women’s health, Sweden

## Abstract

**Background:**

Women are disproportionately diagnosed with symptom-based conditions, notably post-COVID condition (PCC). In Sweden, as of February 2022, 2.3% of PCR-verified female COVID-19 cases versus 1.6% of male cases had a PCC diagnosis. Post-viral fatigue syndrome (PVFS) and exhaustion disorder (ED), a common and relevant diagnosis in Sweden, share substantial symptom overlap with PCC.

**Aims:**

To quantify the association between pre-pandemic, symptom-based primary-care visits and subsequent PCC, PVFS, and ED among adult women, adjusting for risk factors for severe COVID-19.

**Methods:**

We conducted a registry-based prospective cohort study of 208,050 women from the Swedish Medical Birth Register, linked to primary-care data and national sociodemographic registers. The exposure was the frequency of visits for predefined symptom-based conditions during 2015–2019. Adjusted odds ratios (ORs) for diagnoses in 2020–2024 were estimated using logistic regression controlling for BMI, education, age, and region of birth.

**Results:**

Across 2,431,182 primary-care physician visits, 19% were symptom-based. Women with >8 such visits had higher odds of all three outcomes: PCC (OR 5.45, 95% CI 4.43–6.71), PVFS (OR 7.71, 95% CI 5.97–9.96), and ED (OR 5.32, 95% CI 4.84–5.85). Pre-pandemic BMI and education were not associated with PCC or PVFS but showed some association with ED. Still, 17% of women with PCC had no recorded symptom-based visits before the pandemic.

**Conclusions:**

Pre-pandemic symptom-based primary-care visits were strongly associated with higher risk of PCC, PVFS, and ED in a dose-dependent way, but modest discrimination underscores heterogeneous individual risk. Patterns suggest other influences alongside biological susceptibility.

## Introduction

COVID-19 vaccines were developed, tested, and deployed with unprecedented efficiency and speed. By comparison, efforts to identify and evaluate therapies for the persistent symptoms associated with post-COVID condition (PCC) have lagged behind. Despite five years of research, no conclusive definition or set of biomarkers for PCC has been generally accepted [1,2]. More than 200 different symptoms have been associated with the diagnosis. Several mechanisms and explanatory models have been suggested [2,3], but no consistent pattern has emerged. Differences are also evident between individuals with severe disease (e.g. who were hospitalized and/or sustained structural damage from infection), and those who experienced mild disease – or even no apparent symptoms of COVID-19 at all [4,5]. Reported prevalence and cost estimates for PCC vary widely. In a Dutch population-based cohort, 27–64% of SARS-CoV-2–positive adults reported persistent symptoms depending on case definition, with approximately 18–26% attributable to infection when compared with controls. At the same time, highly cited global cost figures (e.g. up to USD 1 trillion annually) are based on economic modelling rather than directly observed expenditure, and vary substantially depending on whether healthcare use, work absence, informal care, and disability are included [6–8].

In October 2020 the code U09.9 for PCC was introduced in Sweden, following the recommendations and criteria from the WHO. Prior to this the codes U08.9 and Z861A for ‘Personal history of COVID-19, unspecified’ were used in combination with a symptom diagnosis. During the pandemic and in the years that followed, most diagnostics and care for PCC were handled within primary-care settings.

The emergence of PCC mirrors earlier known episodes of ‘post viral fatigue’ down to many details – but on a global scale [9]. Complaints and symptoms are usually varying, with patients reporting excessive fatigue, pain, palpitations, reduced fitness, and post-exertional malaise. Usually, there are few objective findings and patients may feel that they are not being taken seriously, with resulting mistrust and dissatisfaction with care [9,10]. As with similar conditions, such as post-viral fatigue syndrome (PVFS) and Sweden’s uniquely defined exhaustion disorder (ED), therapeutic options remain limited. Cognitive behavioural therapy and symptom management represent the few evidence-supported strategies available to date [11].

PVFS, classified under International Classification of Diseases, Tenth Revision (ICD-10) code G93.3, shares many clinical features with PCC [12]. The diagnosis has roots in earlier constructs such as neurasthenia and chronic fatigue syndrome (CFS), with a history of cluster outbreaks and hypothesized but unconfirmed infectious aetiologies [9,13]. ED (ICD-10 F43.8A), introduced in Sweden in 2005, reflects a stress-related syndrome with cognitive, emotional, and physical symptoms. Unlike PCC and PVFS, ED is characterized by identifiable external stressors, often occupational in nature, and lacks a presumed infectious trigger [14].

All three conditions – PCC, PVFS, and ED – are symptom-driven and lack definitive diagnostic tests. Their overlapping presentations raise questions about whether they represent distinct pathologies or variants within a shared clinical spectrum. Furthermore, the high prevalence of these symptoms in the general population complicates the diagnostic process and may result in differential diagnosis based on care-seeking behaviour, sociodemographic characteristics, or provider interpretation rather than distinct underlying pathology. All three conditions are also more common in women [14,15], adding a gender and/or sex aspect to the diagnostic process.

Consultations in primary care for somatic symptoms without an identifiable medical cause are common. Although recent data from Sweden are lacking, a cross-sectional study conducted in Danish primary care found that in 36% of patients no definitive underlying diagnosis could be established [16]. These findings are consistent with earlier studies conducted in the United States [17]. Somatic symptoms frequently co-occur with general psychological distress and nonspecific mental health issues that do not meet the criteria for any defined psychiatric diagnosis. In addition to negatively impacting quality of life and contributing to work disability and elevated healthcare costs, unexplained symptoms are often associated with significant patient dissatisfaction [18].

These patterns suggest that the development of PCC, PVFS, and ED may depend not only on a biological trigger but also on how symptoms are experienced, interpreted, and acted on in primary care. Symptom-based care-seeking may therefore serve as a marker of underlying vulnerability and the clinical pathways through which symptoms become diagnosed. However, this link has not been examined. By analyzing pre-pandemic symptom-based primary-care visits, we can assess whether these diagnoses can be associated with pre-existing utilization patterns and if PCC, PVFS, and ED share similar diagnostic pathways.

The primary aim of this study was to determine the extent to which pre-pandemic symptom-based primary-care visits predict subsequent diagnoses of PCC, and to compare these associations with those for PVFS and ED. We also examined the contribution of known risk factors for severe COVID-19 for the risk of PCC diagnosis and the associations with occupation given its relevance for both viral exposure and psychosocial stress.

## Methods

### Study population and data sources

This is a prospective cohort study, utilizing the database of the Swedish COVID-19 Investigation for Future Insights – a Population Epidemiology Approach using Register Linkage (SCIFI-PEARL) project [19]. Using Sweden’s personal identity number system, national and regional health registers were cross-linked.

The study population of women of childbearing age was defined using the Medical Birth Register (MBR) which provided a large, representative female population. We included all women residing in the Stockholm or Gothenburg metropolitan areas who gave birth between 2014 and 2019, representing approximately 40% of the Swedish female population in this age range.

Additional data sources included the Longitudinal Integrated Database for Health Insurance and Labour Market Studies (LISA), the National Register of Notifiable Diseases (SmiNet), the National Patient Register, and regional primary-care databases in Västra Götaland (VEGA) and Stockholm (VAL).

The MBR, managed by the National Board of Health and Welfare, records antenatal care in all women in Sweden resulting in the birth of a child since 1973 and includes measured weight and height from the first visit (6–12 weeks of gestation) [20]. LISA provides sociodemographic data, including education and income. SmiNet contains records of notifiable communicable diseases, including all SARS-CoV-2 PCR test results. VEGA and VAL cover nearly all primary-care encounters, including visit dates, diagnoses, and provider type.

**Figure 1.**
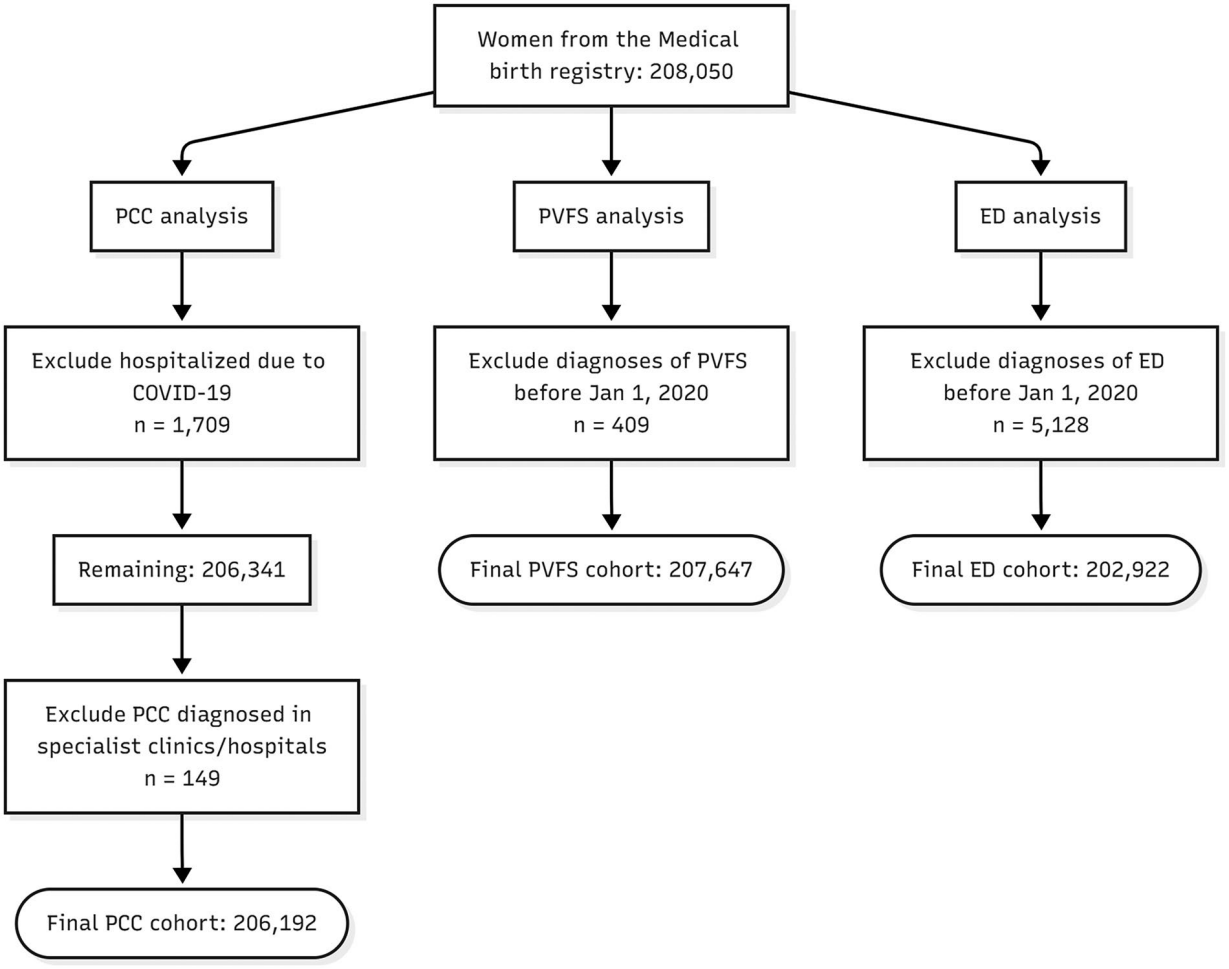
Flow of participants into the analytic samples. This flowchart illustrates the derivation of three analytic samples for the main analysis: post-COVID condition (PCC) in patients not hospitalized for COVID-19, post-viral fatigue syndrome (PVFS), and exhaustion disorder (ED). Starting from the initial cohort of 208,050 women from the Medical Birth Registry, stepwise exclusions were applied for each outcome.

Three analytic cohorts were created from the MBR. The PCC cohort included all eligible women and tracked PCC diagnoses from 2020 to 2024. The PVFS and ED cohorts tracked cases during the same time frame and excluded those with a diagnosis before baseline. PCC diagnoses outside of primary care (hospital or outpatient specialist care) and those hospitalized for COVID-19 were excluded to focus on primary-care managed cases (Figure 1).

### Outcomes

Primary outcomes were binary indicators for a diagnosis of PCC (ICD-10 U09.9), PVFS (ICD-10 G93.3), or ED (ICD-10 F43.8A) between 2020 and 2024 in either VAL or VEGA. Each outcome was evaluated in a distinct cohort, and individuals who were coded in the National Patient Registry as inpatient due to COVID-19 or diagnosed with PCC in specialist outpatient clinics were excluded in the main analysis to focus on less severe cases handled in primary care.

### Main exposure

We adopted a practical strategy for identifying relevant symptoms, selecting ICD-10 codes commonly used in Swedish primary care to denote non-specific or symptom-based conditions. The complete list is provided in Supplementary Table S1. To define what symptoms *actually are* [21,22] falls outside the scope of this paper.

Each participant’s number of visits for symptom-based diagnoses between 1 January 2015 and 1 July 2019, was calculated. These were categorized into six groups: 0, 1–2, 3–4, 5–6, 7–8, and >8 visits. We applied a 6-month exposure–outcome lag to minimize protopathic bias from prodromal care.

### Additional exposures

Obesity and socioeconomic disadvantage reflect underlying health status and have been linked to severe COVID-19 outcomes, although associations with PCC are less clear [4,23]. BMI was derived from early-pregnancy measurements in the MBR and categorized according to WHO (underweight, normal, overweight, obese I, obese II–III) [24].

Education was used as a proxy for socioeconomic position, as well as a social and behavioral determinant that influences both health status and health-care utilization patterns. It was obtained from LISA as of 1 Jan 2020 and classified as low (≤9 years), medium (high school or ≤2 years tertiary/university), and high (≥3 years tertiary/university). Age was computed as of 31 Dec 2019. Country of birth was categorized as Sweden, Europe (except Sweden), Africa, Asia, or the Americas/Oceania. Occupation as of 1 Jan 2020 was obtained from LISA, using the SSYK2 system, based on ISCO-08 (for codes and descriptions, see Supplementary Table S4). These covariates were included to reduce confounding by baseline differences in health status and health-care utilization.

## Statistical methods

The main exposure was the number of symptom-based primary-care visits between 2015 and 2019. We used multivariable logistic regression to calculate adjusted odds ratios (ORs) with 95% confidence intervals (CIs) for associations with each outcome, adjusting for age, BMI, education and region of birth. As missingness was modest (BMI *n* = 12,834, 6.22%; education *n* = 7,267, 3.52%; region of birth *n* = 29, 0.014%), the primary analyses used complete cases. As a robustness check, we repeated models including all participants by adding a ‘missing’ category for each covariate. Estimates were similar, indicating that the findings are not driven by the handling of missing data.

In a complementary analysis, occupation was the exposure. Each category was defined by the corresponding two-digit SSYK2-code (see Supplementary Table S4 for all codes), with all other codes combined as the reference.

Sensitivity analyses included [1]: including those hospitalized with COVID-19 and those with PCC diagnoses made in hospitals and specialist outpatient clinics [2]; excluding individuals with a prior ED (F43.8A) primary-care diagnosis during 2015–2019 from the PCC analysis [3]; removing psychiatric symptoms from the exposure list for all three outcomes [4]; additionally adjusting the PCC model for non-symptom primary-care visits (total visits in 2015–2019 minus symptom-related visits), to account for general healthcare utilization and diagnostic opportunity; and [5] an intersectional analysis among women in care occupations (*n* = 24,927), a group with comparatively lower educational attainment and a higher proportion born outside Sweden, using ED as the outcome due to its higher prevalence.

To assess specificity and residual confounding related to healthcare utilization and symptom reporting, we modelled a negative-control outcome together with two graded positive controls with varying reporting sensitivity: hypothyroidism (ICD-10 E03.9), migraine (ICD-10 G43), and irritable bowel syndrome (ICD-10 K58). All analyses were conducted using SAS version 9.4 (SAS Institute, Cary, NC), with statistical significance set at a two-sided *p*-value <0.05.

## Results

From the population of 208,050 women who had been pregnant between 2014 and 2019 in either Stockholm or Gothenburg, 1,858 were excluded from the main PCC analysis – 1,709 due to COVID-19 hospitalization and 149 due to receiving a PCC diagnosis in specialist care. For the PVFS analysis, 409 women were excluded based on a pre-2020 diagnosis, and for ED, 5,128 were excluded due to a pre-2020 diagnosis (Figure 1).

Across the study population, 8,231 unique diagnostic codes were recorded in primary care between 2015 and 2019. In total, 2,431,182 primary-care physician visits were logged, A small number of codes accounted for most visits: 73 codes comprised 50% of all visits, while 268 codes covered 75%. The most used codes are listed in Supplementary Tables S2–S3. Nineteen per cent of all visits had one of the symptom-based ICD-10 codes used for this study as main diagnosis.

**Table 2.** Multivariable logistic regression estimates.

Variable	Odds ratio PCC (95 %CI)	Odds ratio PVFS (95% CI)	Odds ratio ED (95% CI)
Age	**1.06 (**1.04–1.07)	**1.05** (1.04–1.07)	**1.04** (1.03–1.04)
Region of birth (reference Sweden)			
Europe without Sweden	**1.26** (1.05–1.50)	0.90 (0.70–1.17)	**0.61** (0.56–0.67)
Africa	**0.55** (0.38–0.78)	**0.41** (0.24–0.70)	**0.18** (0.15–0.23)
Asia	0.91 (0.76–1.09)	0.88 (0.69–1.11)	**0.38** (0.34–0.42)
Americas, Oceania	1.14 (0.80–1.62)	1.23 (0.79–1.91)	**0.69** (0.58–0.83)
BMI (reference normal)			
Underweight	1.03 (0.70–1.51)	1.09 (0.67–1.77)	1.18 (1.01–1.38)
Overweight	1.01 (0.89–1.16)	0.92 (0.76–1.10)	1.03 (0.97–1.09)
Obese (class I)	1.00 (0.81–1.22)	0.95 (0.73–1.24)	**1.15** (1.06–1.25)
Obese (class II-III)	1.17 (0.88–1.54)	1.07 (0.74–1.55)	1.08 (0.96–1.23)
Education (Reference low)			
Medium	1.17 (0.92–1.49)	1.01 (0.75–1.38)	**1.33** (1.19–1.48)
High	1.16 (0.91–1.48)	1.10 (0.81–1.50)	**1.27** (1.13–1.42)

*Note*: Age modelled per 1-year increase. Reference categories: place of birth = Sweden; BMI = normal (early pregnancy); education = low (≤9 years). All estimates mutually adjusted for age, BMI, education, and region of birth. Analytic samples correspond to those described in Table 1. Odds ratios (95% confidence intervals) from logistic regression models estimating associations between covariates and subsequent diagnosis of post covid condition (PCC), post-viral fatigue syndrome (PVFS), or exhaustion disorder (ED) during 2020–2024. Boldface indicates odds ratios with 95% confidence intervals that do not include 1.0.

Women with a PCC, PVFS, or ED diagnosis tended to be slightly older, more educated and more often born in Sweden or elsewhere in Europe than the total study population. They also had a higher frequency of both total and symptom-based primary-care visits prior to the pandemic (Table 1). However, a substantial proportion of diagnosed women had no recorded symptom-based visits before the pandemic – 17% for PCC, 15% for PVFS, and 11% for ED.

**Table 1.** Baseline characteristics of cohort.

Characteristic	Total population	PCC diagnosed	PVFS diagnosed	ED diagnosed
*N*	208,050	1,304	753	7,550
Age in 2020 (mean)	33.8	35.5	35.3	35.1
Education				
Low	8.3%	6.2%	7.3%	4.9%
Medium	44.4%	45.1%	45.2%	47.2%
High	43.6%	47.4%	46.5%	47.5%
Missing	3.5%			
Region of birth				
Sweden	67.5%	71.2%	74.7%	84.0%
Europe without Sweden	10.9%	12.1%	9.5%	7.2%
Africa	5.6%	2.8%	2.7%	1.0%
Asia	13.8%	11.4%	10.3%	6.0%
Americas/Oceania	2.2%	2.5%	2.8%	1.8%
Missing	0.01%			
BMI Category				
Normal	55.3%	54.1%	56.1%	53.7%
Overweight	24.0%	23.8%	22.9%	23.8%
Obese I	8.6%	8.5%	8.7%	9.5%
Obese II–III	3.5%	3.8%	3.2%	4.0%
Missing	6.2%			
Primary care physician visits				
Mean total visits (2015–2019)	12.7	20.5	22.3	18.7
Mean symptom-based visits	2.2	4.5	5.3	3.6
Symptom-based visit categories				
0 visits	36.8%	17.7%	15.5%	11.7%
1–2 visits	34.6%	30.4%	27.5%	22.9%
3–4 visits	13.9%	18.6%	17.9%	17.5%
5–6 visits	6.5%	12.6%	11.0%	12.8%
7–8 visits	3.2%	6.4%	6.2%	8.7%
>8 visits	5.0%	14.4%	21.9%	26.4%

*Note*: Education measured as of 1 Jan 2020 (low ≤9 years; medium high school/≤2 years tertiary; high ≥3 years tertiary). BMI categories are based on early pregnancy measurements. Primary-care physician visits recorded 2015–2019; symptom-based visits defined using ICD-10 codes (see Supplementary Table S1). Diagnostic outcomes assessed 2020–2024. Percentages may not sum to 100 due to rounding. Baseline sociodemographic, anthropometric, and healthcare utilization characteristics of the total study population and subgroups subsequently diagnosed with post-Covid condition (PCC), post-viral fatigue syndrome (PVFS), or exhaustion disorder (ED). Values are reported as means or percentages.

There was considerable overlap between the diagnoses; of those with PCC, 20% had a diagnosis of ED and 11% of PVFS between 2015 and 2024. Forty-eight individuals had all three diagnoses in 2024. If only diagnoses between 2015 and 2019 were counted the overlap was smaller; of those with PCC, 8% had an earlier diagnosis of ED and 0.3% of PVFS.

**Figure 2.**
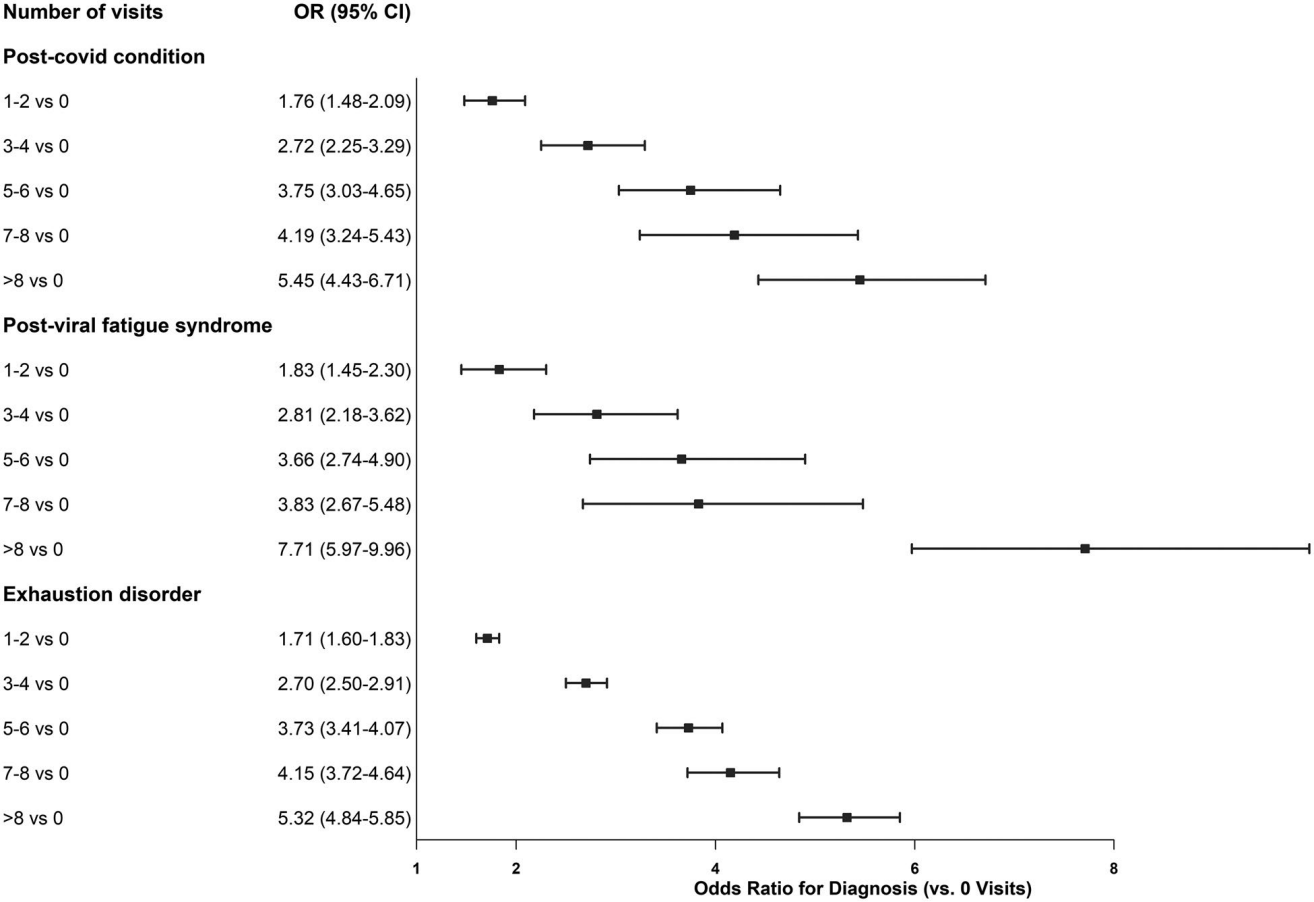
Adjusted odds ratios (95% CIs) for subsequent diagnosis of post-COVID condition (PCC), post-viral fatigue syndrome (PVFS), and exhaustion disorder (ED) during 2020–2024 among adult Swedish women. Analytic sample sizes differ by outcome: PCC *n* = 206,192; PVFS *n* = 207,647; ED *n* = 202,922. Estimates compare categories of pre-pandemic symptom-based primary-care visits (1–2, 3–4, 5–6, 7–8, >8) with 0 visits and are adjusted for age, BMI at baseline (early pregnancy), education (as of 1 Jan 2020), and region of birth. The vertical reference line indicates OR = 1.

Figure 2 shows adjusted odds ratios from logistic regression models for the three diagnostic outcomes. A clear dose-response relationship was evident, with more symptom-related visits between 2015 and mid-2019 associated with increased odds of any of the prespecified diagnoses. AUC values for the models were moderate, ranging from 0.68 to 0.69.

Among the covariates included in the models for adjustment, BMI and education had minimal association with PCC or PVFS (Table 2). However, Class I obesity was weakly associated with increased odds of ED. Higher education also correlated modestly with higher ED risk. Individuals born in Africa had significantly lower odds for all three diagnoses compared to those born in Sweden. For ED, all foreign-born groups had lower odds compared to Swedish-born individuals. This finding was also evident in the results of sensitivity analysis 5. Using ED as the outcome (*n* = 1,088), we observed a similar, and in fact stronger, reduction of odds ratios among women born in Africa in care occupations without higher education (OR 0.11, 95% CI 0.07–0.17), compared with the full population estimate (OR 0.18, 95% CI 0.14–0.22).

Analyses in the subgroup of women with occupational information (profession code available for 83% of the population) showed elevated odds of PCC for highly educated healthcare workers (OR 1.6, 95% CI 1.3–1.9), compared to the whole population, teachers and preschool staff (OR 1.5, 95% CI 1.3–1.8), and care workers without higher education (OR 1.4, 95% CI 1.2–1.6). Teachers, engineers, transport workers, and social workers showed increased odds for ED, too, while cleaners had the lowest odds (OR 0.34, 95% CI 0.3–0.9). For PVFS, elevated risks were found among social workers and highly educated healthcare professionals compared to the whole population. The effect size for occupational results were slightly reduced when controlled for number of visits, BMI, age and country of birth. A list of code frequency is found in Table S4, codes with significant association with PCC, PVFS and ED are found in Tables S5–S7.

Sensitivity analyses demonstrated robust associations across expanded definitions and alternative exposure sets. Including those hospitalized with COVID-19 (*n* = 1,709) as well as diagnosed with PCC in specialist outpatient clinics or hospital (*n* = 149) maintained the association (Table S8). Excluding those diagnosed with ED during 2015–2019 (*n* = 5,128) slightly lowered the estimate (Table S9). Removing psychiatric symptom diagnoses from the baseline symptom list increased ORs for PCC and PVFS while slightly decreasing them for ED (Table S10).

After additionally adjusting for non-symptom primary-care visits, estimates attenuated but the dose–response persisted (PCC OR (>8 vs 0): 4.41 vs 5.45 in the primary model) (Table S11).

Associations with hypothyroidism were substantially weaker and showed a flat dose–response across prior symptom-visit categories. In contrast, associations were stronger for migraine (G43) and strongest for IBS (K58). When further adjusted for the total number of primary-care physician visits during 2014–2019, the estimates for hypothyroidism attenuated to the null (ORs ≈1 across categories), whereas associations for migraine and IBS remained (Table S12).

## Discussion

This study shows that pre-pandemic primary-care visits for symptom-based conditions were strongly associated with later diagnoses of PCC, PVFS, and ED in a dose-dependent fashion. Yet a subset of diagnosed women had little or no prior primary-care contact, underscoring heterogeneity. Consistent with this, discriminatory accuracy was modest (AUC ≈0.68–0.69): the models distinguished cases from non-cases better than chance but are not suitable for individual prediction. These AUCs suggest that prior care-seeking captures a meaningful, though incomplete, risk signal: informative for population-level planning and etiologic inference, while individual risk remains determined by biological, psychosocial, and system-level factors. The observed diagnostic overlap further supports the interpretation that these conditions emerge within a partially shared clinical and diagnostic context, rather than as fully distinct disease entities.

Sensitivity analyses strengthened these conclusions. Including PCC cases from hospital and specialist care produced similar associations, arguing against effects of care setting or disease severity. Excluding women with pre-2020 ED only slightly reduced the estimates, suggesting some diagnostic overlap but not as the main driver. Removing psychiatric symptom codes from the exposure increased the associations for PCC and PVFS but slightly reduced the association for ED, suggesting that ED is more closely linked to pre-existing psychiatric symptom presentations, while PCC and PVFS are less so. Further adjustment for total primary-care visits attenuated estimates but preserved a clear dose–response, indicating that general utilization does not fully explain the associations. Thus, symptom-based visits may reflect a broader illness behaviour pattern related to chronic stress, bodily vigilance, and vulnerability to persistent post-trigger symptoms.

The combined negative/positive-control analyses showed weak and flat associations for hypothyroidism, a laboratory-confirmed condition, but increasingly strong associations for migraine and IBS, which rely more on symptom-based clinical interpretation. This gradient supports a biopsychosocial understanding, where post-trigger symptoms are influenced not only by the initiating event but also by pre-existing symptom patterns and the diagnostic context as well as indicating that diagnostic pathways involving symptom perception and clinical interpretation may play a greater role where reporting sensitivity is higher.

Women born in Africa were underrepresented among PCC cases despite higher risk of severe COVID-19 [25], similar or stronger patterns appeared for PVFS and ED. This pattern persisted even within care occupations, where socioeconomic position and workload are more similar. This suggests that the difference is not explained by socioeconomic status or occupational exposure alone, but reflects culturally patterned ways of recognizing, expressing, and seeking care for persistent symptoms, as well as how clinicians categorize and legitimize non-specific syndromes in primary care. In this view, the trigger (infection or prolonged stress) may be similar across groups, while the pathways through which symptoms become named and treated as PCC, PVFS, or ED are culturally mediated [11]. A Swedish PCC study in specialist setting reporting higher risk of PCC among African-born individuals further illustrates that diagnostic setting and clinical framing shape who receives these labels [26].

Certain occupational groups, particularly teachers and preschool teachers and health care personnel, were overrepresented among those diagnosed. Two plausible explanations for this emerge. First, these professions likely faced higher exposure to SARS-CoV-2 due to limited possibilities for remote work, and accumulating evidence suggests that repeated infections may increase the risk of developing PCC. They were likely also infected earlier, with less protection from vaccine. Second, teachers are also overrepresented in exhaustion disorder statistics, pointing to roles characterized by high demands, low autonomy, and increasing psychosocial stress. Disentangling the effects of viral exposure from these underlying psychosocial stressors remains challenging, especially given the significant symptomatic overlap between PCC and exhaustion disorder. This reflects broader societal dynamics, particularly the ways in which social expectations shape illness experiences. The symptoms commonly reported by PCC patients align with cultural narratives surrounding high-achieving women who ultimately experience collapse under cumulative pressure which has been a recurrent theme in the media [27,28].

The study has several limitations. Our population was primarily urban, limiting generalizability to rural areas with greater physical distance to primary healthcare. Moreover, pregnancy at baseline introduced potential confounding factors, as both healthcare utilization and life circumstances change markedly during pregnancy and the postnatal period, potentially influencing symptom reporting and diagnosis patterns. Another challenge concerns the use of the symptom-based diagnostic codes selected. Focusing solely on these poses the risk of excluding other frequent healthcare users who do not receive these exact diagnoses. As mentioned earlier, more than 8,000 ICD-10 codes were used during follow-up, and the associations might change if more codes were included in the analysis. The combination of codes used in this study do show stronger effects than any specific symptom, such as fatigue or cough [29,30].

It is well-established that women utilize primary-care services somewhat more frequently than men [31,32], a fact that may partly account for the higher incidence of PCC diagnoses observed in women [15]. Increased healthcare contact facilitates the accumulation of diagnoses, which eventually could result in umbrella terms such as PCC that encapsulate diverse symptoms. This phenomenon creates a feedback loop wherein greater healthcare use generates further diagnostic labelling, thereby reinforcing continued healthcare-seeking behaviour [29]. This discussion also intersects with the existence of the term exhaustion disorder, which in the Swedish context functions as a broad diagnostic label that often reflects adverse working or living conditions rather than a distinct biological pathology. Future studies that include men are warranted to assess generalizability and to disentangle sex- and gender-specific pathways, especially given men’s different COVID-19 severity profiles, occupational exposures, and healthcare-seeking patterns.

## Conclusions

Pre-pandemic primary-care visits for symptom-based conditions were strongly associated with later PCC, PVFS, and ED. These associations were robust to multiple sensitivity checks and not explained by general healthcare utilization. Yet modest discriminatory accuracy highlights substantial individual heterogeneity.

Taken together, the findings support a biopsychosocial interpretation: a trigger (infection or prolonged stress) may initiate symptoms, but illness behavior, stress exposure, symptom interpretation, and diagnostic practices shape which individuals develop persistent symptom syndromes and receive these diagnoses. The underrepresentation of women born in Africa indicate that cultural contexts influence pathways into diagnosis. Future research should integrate biological, psychosocial, and healthcare-system approaches to understand persistence, recovery, and equity in care.

## Supplementary Material

Supplementary_Materials review 2.docx

## Data Availability

The individual-level register data used in this study are held by Swedish authorities and cannot be shared publicly under Swedish law. Aggregated data and the statistical code used for analysis are available from the corresponding author upon reasonable request and with necessary permissions from data owners.

## References

[1] Liew F, Efstathiou C, Fontanella S, et al. Large-scale phenotyping of patients with long COVID post-hospitalization reveals mechanistic subtypes of disease. Nat Immunol. 2024;25(4):607–621. doi: 10.1038/s41590-024-01778-0. 38589621 PMC11003868

[2] Peluso MJ, Deeks SG. Mechanisms of long COVID and the path toward therapeutics. Cell. 2024;187(20):5500–5529. doi: 10.1016/j.cell.2024.07.054. 39326415 PMC11455603

[3] Raijmakers RPH, Lund Berven L, Keijmel SP, et al. Immunological associations in post-infective fatigue syndromes including Long COVID—a systematic review and meta-analysis. eBioMedicine. 2025;6121:105970. doi: 10.1016/j.ebiom.2025.105970. 41151241 PMC12596658

[4] Tsampasian V, Elghazaly H, Chattopadhyay R, et al. Risk factors associated with post − COVID-19 condition: a systematic review and meta-analysis. JAMA Intern Med. 2023;183(6):566–580. doi: 10.1001/jamainternmed.2023.0750. 36951832 PMC10037203

[5] Selvakumar J, Havdal LB, Brodwall EM, et al. Risk factors for fatigue severity in the post-COVID-19 condition: a prospective controlled cohort study of nonhospitalised adolescents and young adults. Brain Behav Immun Health. 2025;44:100967. doi: 10.1016/j.bbih.2025.100967. 40094121 PMC11908541

[6] Al-Aly Z, Davis H, McCorkell L, et al. Long COVID science, research and policy. Nat Med. 2024;30(8):2148–2164. doi: 10.1038/s41591-024-03173-6. 39122965

[7] Høeg TB, Ladhani S, Prasad V. How methodological pitfalls have created widespread misunderstanding about long COVID. BMJ Evid Based Med. 2024;29(3):142–146. doi: 10.1136/bmjebm-2023-112338. PMC11137465 37748921

[8] Pagen DME, van Bilsen CJA, Brinkhues S, et al. Prevalence of long-term symptoms varies when using different post-COVID-19 definitions in positively and negatively tested adults: the PRIME post-COVID study. Open Forum Infect Dis. 2023;10(10):ofad471. doi: 10.1093/ofid/ofad471. 37885796 PMC10599319

[9] Aronowitz RA. The trouble with chronic fatigue. J Gen Intern Med. 1991;6(4):378–379. doi: 10.1007/BF02597441. 1819250

[10] Johannisson K. Modern fatigue: a historical perspective. In: Stress in health and disease. Hoboken (NJ): John Wiley & Sons, Ltd; 2006. p. 1–19.

[11] Zeraatkar D, Ling M, Kirsh S, et al. Interventions for the management of long covid (post-covid condition): living systematic review. BMJ. 2024;387:e081318. doi: 10.1136/bmj-2024-081318. 39603702 PMC11600537

[12] Nimnuan C, Rabe-Hesketh S, Wessely S, et al. How many functional somatic syndromes? J Psychosom Res. 2001;51(4):549–557. doi: 10.1016/s0022-3999(01)00224-0. 11595242

[13] Cortes Rivera M, Mastronardi C, Silva-Aldana CT, et al. Myalgic encephalomyelitis/chronic fatigue syndrome: a comprehensive review. Diagnostics. 2019;9(3):91. doi: 10.3390/diagnostics9030091. 31394725 PMC6787585

[14] Glise K, Ahlborg G, Jonsdottir IH. Prevalence and course of somatic symptoms in patients with stress-related exhaustion: does sex or age matter. BMC Psychiatry. 2014;14(1):118. doi: 10.1186/1471-244X-14-118. 24755373 PMC3999732

[15] Bygdell M, Leach S, Lundberg L, et al. A comprehensive characterization of patients diagnosed with post-COVID-19 condition in Sweden 16 months after the introduction of the International Classification of Diseases Tenth Revision diagnosis code (U09.9): a population-based cohort study. Int J Infect Dis. 2023;126:104–113. doi: 10.1016/j.ijid.2022.11.021. 36410693 PMC9678230

[16] Rosendal M, Carlsen AH, Rask MT, et al. Symptoms as the main problem in primary care: a cross-sectional study of frequency and characteristics. Scand J Prim Health Care. 2015;33(2):91–99. doi: 10.3109/02813432.2015.1030166. 25961812 PMC4834508

[17] Katon WJ, Walker EA. Medically unexplained symptoms in primary care. J Clin Psychiatry. 1998;59(suppl 20):11470. 9881537

[18] Kroenke K. A practical and evidence-based approach to common symptoms. Ann Intern Med. 2014;161(8):579–586. doi: 10.7326/M14-0461. 25329205

[19] Nyberg F, Franzén S, Lindh M, et al. Swedish Covid-19 investigation for future insights: a population epidemiology approach using register linkage (SCIFI-PEARL). Clin Epidemiol. 2021;13:649–659. doi: 10.2147/CLEP.S312742. 34354377 PMC8331198

[20] Cnattingius S, Källén K, Sandström A, et al. The Swedish medical birth register during five decades: documentation of the content and quality of the register. Eur J Epidemiol. 2023;38(1):109–120. doi: 10.1007/s10654-022-00947-5. 36595114 PMC9867659

[21] Eriksen TE, Risør MB. What is called symptom? Med Health Care Philos. 2014;17(1):89–102. doi: 10.1007/s11019-013-9501-5. 23877313

[22] Malterud K, Guassora AD, Graungaard AH, et al. Understanding medical symptoms: a conceptual review and analysis. Theor Med Bioeth. 2015;36(6):411–424. doi: 10.1007/s11017-015-9347-3. 26597868

[23] Peters SAE, MacMahon S, Woodward M. Obesity as a risk factor for COVID-19 mortality in women and men in the UK biobank: comparisons with influenza/pneumonia and coronary heart disease. Diabetes Obes Metab. 2021;23(1):258–262. doi: 10.1111/dom.14199. 32969132 PMC7536945

[24] Inskip H, Crozier S, Baird J, et al. Measured weight in early pregnancy is a valid method for estimating pre-pregnancy weight. J Dev Orig Health Dis. 2021;12(4):561–569. doi: 10.1017/S2040174420000926. 33046167

[25] Rostila M, Cederström A, Wallace M, et al. Disparities in coronavirus disease 2019 mortality by country of birth in Stockholm, Sweden: a total-population-based cohort study. Am J Epidemiol. 2021;190(8):1510–1518. doi: 10.1093/aje/kwab057. 33710317 PMC7989658

[26] Cederström A, Mkoma GF, Benfield T, et al. Long COVID and its risk factors in migrants: a nationwide register study from Sweden. BMC Med. 2025;23(1):53. doi: 10.1186/s12916-025-03900-x. 39875996 PMC11776292

[27] What exactly is long covid? New Eng J Med. 2024;391(10):e17. 39259895 10.1056/NEJMp2407614

[28] Yong E. Long COVID is being erased—again [Internet]. *The Atlantic*; 2023. [cited 2025 Apr 3]. Available from: https://www.theatlantic.com/health/archive/2023/04/long-covid-symptoms-invisible-disability-chronic-illness/673773/.

[29] Reme BA, Gjesvik J, Magnusson K. Predictors of the post-COVID condition following mild SARS-CoV-2 infection. Nat Commun. 2023;14(1):5839. doi: 10.1038/s41467-023-41541-x. 37730740 PMC10511472

[30] Lak V, Sjöland H, Adiels M, et al. Preexisting symptoms increase the risk of developing long COVID during the SARS-CoV-2 pandemic. J Intern Med. 2025;298(2):107–122. doi: 10.1111/joim.20102. 40464158 PMC12239058

[31] Ballering AV, Olde Hartman TC, Verheij R, et al. Sex and gender differences in primary care help-seeking for common somatic symptoms: a longitudinal study. Scand J Prim Health Care. 2023;41(2):132–139. doi: 10.1080/02813432.2023.2191653. 36995265 PMC10193899

[32] Osika Friberg I, Krantz G, Määttä S, et al. Sex differences in health care consumption in Sweden: a register-based cross-sectional study. Scand J Public Health. 2016;44(3):264–273. doi: 10.1177/1403494815618843. 26647097

