# Supplementary material for: Pre-pandemic care-seeking patterns and subsequent diagnoses of post-COVID condition, post viral fatigue syndrome, and exhaustion disorder: a registry-based cohort study of 208,050 Swedish women
Source: Scand J Prim Health Care. 2026 Jan 6;44(1):2611886. doi: 10.1080/02813432.2025.2611886 (PMC12781934; doi:10.1080/02813432.2025.2611886)
Supplement: Supplementary_Materials review 2.docx [file IPRI_A_2611886_SM5482.docx]

**Pre-pandemic care-seeking patterns and subsequent diagnoses of post-COVID condition, post viral fatigue syndrome, and exhaustion disorder among Swedish women**

Authors: Agnes af Geijerstam, Kirsten Mehlig, Fredrik Nyberg, Annika Rosengren, Ailiana Santosa, Maria Åberg, Lauren Lissner

**Supplementary materials**

**Contents**

Page:

2: **Table S1.** ICD-10 codes used to define the exposure set of “symptom-based” diagnoses in primary care (grouped across mental health, pain, respiratory, gastrointestinal, dermatologic, sleep, and general symptom codes). These codes were applied to visits during 2015–2019.

4: **Table S2.** Top primary-care visit diagnoses in the study population, 2015–2019: visit-level counts and ICD-10 definitions, ranked by total number of physician visits.

5: **Table S3.** Most common primary-care diagnoses counted once per individual (person-level frequency) in the study population, with ICD-10 descriptions.

6: **Table S4.** SSYK2 occupational groups present in the cohort (N=156,998 with occupation), with English descriptions and population frequencies (% of those with available data).

8: **Table S5.** SSYK2 occupations with statistically significant associations with post-COVID condition (PCC); odds ratios (OR), 95% CIs, and p-values shown relative to all other occupations.

9: **Table S6.** SSYK2 occupations with statistically significant associations with post-viral fatigue syndrome (PVFS); ORs, 95% CIs, and p-values relative to all other occupations.

10: **Table S7.** SSYK2 occupations with statistically significant associations with exhaustion disorder (ED); ORs, 95% CIs, and p-values relative to all other occupations; groups with lower odds are also indicated.

11: **Table S8.** Sensitivity analysis expanding the PCC case definition to include women hospitalized with COVID-19 and/or diagnosed with PCC in specialist outpatient or hospital settings; multivariable ORs with 95% CIs.

12: **Table S9.** Sensitivity analysis excluding individuals with an ED diagnosis recorded during 2014–2020; multivariable ORs with 95% CIs for PCC.

13: **Table S10.** Sensitivity analysis removing all psychiatric symptom F-codes from the exposure list; adjusted ORs (95% CIs) for PCC, PVFS (G93.3), and ED across covariates and symptom-visit categories.

14: **Table S11.** Sensitivity analysis for PCC additionally controlling for the number of non-symptom primary-care physician visits in 2014–2019; ORs (95% CIs) by symptom-based visit category (exposure) versus 0 visits.

15: **Table S12.** Negative-control outcome analysis: hypothyroidism (ICD-10 E03.9) diagnosed during 2020–2024. Shows ORs (95% CIs) by symptom-visit category, with and without additional adjustment for non-symptom physician visits to assess residual confounding by healthcare utilization.

16: **Strobe checklist**

# Table S1. ICD-10 codes used to define the exposure set of “symptom-based” diagnoses in primary care (grouped across mental health, pain, respiratory, gastrointestinal, dermatologic, sleep, and general symptom codes). These codes were applied to visits during 2015–2019.

| ICD-10 code | Definition |
| --- | --- |
| B349 | Viral infection, unspecified |
| F329 | Depressive episode, unspecified |
| F412 | Mixed anxiety and depressive disorder |
| F413 | Other mixed anxiety disorders |
| F419 | Anxiety disorder, unspecified |
| F430 | Acute stress reaction |
| F432 | Adjustment disorders |
| F438 | Other reactions to severe stress |
| F439 | Reaction to severe stress, unspecified |
| F438A | Exhaustion disorder |
| F454 | Persistent somatoform pain disorder |
| F510 | Nonorganic insomnia |
| F519 | Nonorganic sleep disorder, unspecified |
| G479 | Sleep disorder, unspecified |
| H920 | Otalgia |
| H931 | Tinnitus |
| J018 | Acute sinusitis, unspecified |
| J019 | Acute sinusitis, unspecified |
| J069 | Acute upper respiratory infection, unspecified |
| J300 | Vasomotor rhinitis |
| K309 | Gastritis, unspecified |
| K581 | Irritable bowel syndrome with diarrhea |
| K582 | Irritable bowel syndrome with constipation |
| K583 | Irritable bowel syndrome with mixed bowel habits |
| K589 | Irritable bowel syndrome without diarrhea |
| K590 | Constipation |
| K58 | Irritable bowel syndrome |
| L299 | Pruritus, unspecified |
| L309 | Dermatitis, unspecified |
| L509 | Urticaria, unspecified |
| L659 | Alopecia, unspecified |
| M255 | Joint pain |
| M542 | Cervicalgia |
| M546 | Pain in thoracic spine |
| M549 | Dorsalgia, unspecified |
| M796 | Soft tissue disorder, unspecified |
| M797 | Fibromyalgia |
| N943 | Dysmenorrhea, unspecified |
| R000 | Tachycardia, unspecified |
| R002 | Palpitations |
| R040 | Epistaxis |
| R059 | Cough, unspecified |
| R060 | Dyspnea |
| R05 | Cough |
| R070 | Chest pain, unspecified |
| R073 | Other chest pain |
| R074 | Chest pain, unspecified |
| R104 | Other and unspecified abdominal pain |
| R129 | Nausea and vomiting |
| R219 | Localized swelling, mass and lump, unspecified |
| R238 | Other skin changes |
| R400 | Somnolence |
| R429 | Unspecified symptoms and signs involving cognitive functions and awareness |
| R529 | Pain, unspecified |
| R530 | Fatigue |
| R539 | Malaise and fatigue, unspecified |
| R559 | Unspecified convulsions |
| Z000 | General medical examination |
| Z018 | Encounter for other specified special examinations |
| Z033 | Observation for suspected mental and behavioral disorders |
| Z038 | Observation for other suspected diseases and conditions |
| Z039 | Observation for unspecified suspected disease or condition |
| Z733 | Stress, not elsewhere classified |

# Table S2. Top primary-care visit diagnoses in the study population, 2015–2019: visit-level counts and ICD-10 definitions, ranked by total number of physician visits.

| Obs | ICD-10 code | Count | Definition |
| --- | --- | --- | --- |
| 1 | R104X | 155 622 | Other and unspecified abdominal pain |
| 2 | J069 | 132 297 | Acute upper respiratory infection, unspecified |
| 3 | E039 | 127 075 | Hypothyroidism, unspecified |
| 4 | F438A | 116 530 | Exhaustion disorder/clinical burnout |
| 5 | F419 | 96 627 | Anxiety disorder, unspecified |
| 6 | R539 | 95 794 | Unspecified malaise and fatigue |
| 7 | F412 | 95 621 | Mixed anxiety and depressive disorder |
| 8 | O268 | 83 250 | Other specified pregnancy-related conditions |
| 9 | N979 | 82 362 | Female infertility, unspecified |
| 10 | R529 | 79 015 | Unspecified pain |
| 11 | DIAEJ | 74 194 | No diagnosis |
| 12 | Z349 | 70 385 | Supervision of high-risk pregnancy, unspecified |
| 13 | N300 | 60 692 | Cystitis |
| 14 | O267 | 55 062 | Other venous complications in pregnancy |
| 15 | R519 | 53 043 | Headache, unspecified |
| 16 | R059 | 52 759 | Cough, unspecified |
| 17 | F329 | 50 237 | Major depressive disorder, single episode, unspecified |
| 18 | Z718 | 49 846 | Other specified counseling |
| 19 | Z038 | 48 027 | Observation for suspected disease or condition, unspecified |
| 20 | J459 | 44 292 | Asthma, unspecified |

# Table S3. Most common primary-care diagnoses counted once per individual (person-level frequency) in the study population, with ICD-10 descriptions.

| Obs | ICD-10 code | Count | ICD-10 English Description |
| --- | --- | --- | --- |
| 1 | Z392 | 135 202 | Postpartum care and examination |
| 2 | J069 | 93 839 | Acute upper respiratory infection, unspecified |
| 3 | R529 | 90 631 | Unspecified pain |
| 4 | R104X | 78 270 | Other and unspecified abdominal pain |
| 5 | Z349 | 77 379 | Supervision of high-risk pregnancy, unspecified |
| 6 | R539 | 67 923 | Unspecified malaise and fatigue |
| 7 | Z719 | 57 988 | Counseling, unspecified |
| 8 | R059 | 53 674 | Cough, unspecified |
| 9 | F419 | 50 345 | Anxiety disorder, unspecified |
| 10 | O268 | 47 398 | Other specified pregnancy-related conditions |
| 11 | R519 | 44 127 | Headache, unspecified |
| 12 | F438A | 43 727 | Other venous complications in pregnancy |
| 13 | N300 | 42 609 | Cystitis |
| 14 | Z718 | 40 548 | Other specified counseling |
| 15 | R238 | 38 229 | Other abnormal findings on examination |
| 16 | M545 | 37 850 | Low back pain |
| 17 | B349 | 36 498 | Viral infection, unspecified |
| 18 | M549 | 36 376 | Dorsalgia, unspecified |
| 19 | R219 | 36 233 | Chest pain, unspecified |
| 20 | Z038 | 36 174 | Observation for suspected disease or condition, unspecified |

# Table S4. SSYK2 occupational groups present in the cohort (N=156,998 with occupation), with English descriptions and population frequencies (% of those with available data).

| SSYK2-code | n in population (%) | English Description |
| --- | --- | --- |
| 11 | 258 (0.2%) | Politicians, chief executives and senior officials, etc. |
| 12 | 3 539 (2.3%) | Managers in finance, human resources, marketing and sales, and other administration, etc. |
| 13 | 1 335 (0.9%) | Managers in IT, logistics, R&D, real estate, construction and engineering, and manufacturing, etc. |
| 14 | 347 (0.2%) | Managers in education |
| 15 | 1 274 (0.8%) | Managers in health care and other community/public services |
| 16 | 217 (0.1%) | Managers in banking, finance and insurance |
| 17 | 869 (0.6%) | Managers in other service industries |
| 21 | 6 910 (4.4%) | Occupations requiring advanced higher education in science and engineering |
| 22 | 12 859 (8.2%) | Occupations requiring advanced higher education in health care |
| 23 | 14 642 (9.3%) | Occupations requiring advanced higher education in education |
| 24 | 13 979 (8.9%) | Occupations requiring advanced higher education in economics and administration |
| 25 | 3 200 (2.0%) | Occupations requiring advanced higher education in IT |
| 26 | 8 294 (5.3%) | Occupations requiring advanced higher education in law, culture and social work, etc. |
| 31 | 2 190 (1.4%) | Occupations requiring higher education or equivalent in technology |
| 32 | 1 786 (1.1%) | Occupations requiring higher education or equivalent in health care and laboratory work |
| 33 | 13 360 (8.5%) | Occupations requiring higher education or equivalent in economics and administration |
| 34 | 2 723 (1.7%) | Occupations requiring higher education or equivalent in culture, wellness and social work |
| 35 | 879 (0.6%) | Occupations requiring higher education or equivalent in IT, audio and lighting technology, etc. |
| 41 | 8 934 (5.7%) | Office assistants and secretaries |
| 42 | 3 497 (2.2%) | Customer service occupations |
| 43 | 1 410 (0.9%) | Occupations in materials management, etc. |
| 44 | 313 (0.2%) | Other office and customer service occupations |
| 51 | 4 546 (2.9%) | Service occupations |
| 52 | 9 707 (6.2%) | Sales occupations in retail trade, etc. |
| 53 | 27094 (17.3%) | Care occupations |
| 54 | 755 (0.5%) | Other security and surveillance occupations |
| 61 | 571 (0.4%) | Agriculture and horticulture occupations |
| 62 | 24 (0.0%) | Forestry workers, aquaculture workers and fishers |
| 71 | 465 (0.3%) | Construction and civil engineering occupations |
| 72 | 601 (0.4%) | Metal craft and repair occupations |
| 73 | 167 (0.1%) | Precision mechanics, graphic and arts/craft occupations |
| 74 | 125 (0.1%) | Installation and service occupations in electrical and electronics |
| 75 | 194 (0.1%) | Other craft occupations in wood and textiles, etc. |
| 76 | 217 (0.1%) | Craft occupations in food production |
| 81 | 907 (0.6%) | Process and machine operators |
| 82 | 571 (0.4%) | Assemblers |
| 83 | 683 (0.4%) | Transport and machine-operating occupations |
| 91 | 4 257 (2.7%) | Cleaning occupations |
| 92 | 52 (0.0%) | Berry pickers and planters, etc. |
| 93 | 308 (0.2%) | Other occupations in construction, manufacturing and goods handling |
| 94 | 2 399 (1.5%) | Fast-food workers, kitchen and restaurant assistants, etc. |
| 95 | 24 (0.0%) | Market and street vendors |
| 96 | 452 (0.3%) | Recycling workers, newspaper distributors and other service workers |
| 01 | 15 (0.0%) | Officers |
| 02 | 27 (0.0%) | Specialist officers |
| 03 | 22 (0.0%) | Soldiers, etc. |

# Table S5. SSYK2 occupations with statistically significant associations with post-COVID condition (PCC); odds ratios (OR), 95% CIs, and p-values shown relative to all other occupations.

| SSYK2-code | Odds Ratio | 95% CI low | 95% CI high | p-value | English Description |
| --- | --- | --- | --- | --- | --- |
| 72 | 2.0 | 1.0 | 3.9 | 0.04 | Metal craft and repair occupations |
| 22 | 1.6 | 1.3 | 1.9 | <.0001 | Occupations requiring advanced higher education in healthcare |
| 23 | 1.5 | 1.3 | 1.8 | <.0001 | Occupations requiring advanced higher education in education |
| 53 | 1.4 | 1.2 | 1.6 | <.0001 | Care occupations |
| 25 | 0.5 | 0.3 | 0.9 | 0.02 | Occupations requiring advanced higher education in IT |

# Table S6. SSYK2 occupations with statistically significant associations with post-viral fatigue syndrome (PVFS); ORs, 95% CIs, and p-values relative to all other occupations.

| SSYK2-code | Odds Ratio | 95% CI low | 95% CI high | p-value | English Description |
| --- | --- | --- | --- | --- | --- |
| 26 | 1.4 | 1.0 | 1.9 | 0.03 | Law, culture and social work (advanced education) |
| 22 | 1.3 | 1.0 | 1.7 | 0.04 | Advanced higher education in healthcare |
| 42 | 0.4 | 0.2 | 1.0 | 0.04 | Customer service occupations |

# Table S7. SSYK2 occupations with statistically significant associations with exhaustion disorder (ED); ORs, 95% CIs, and p-values relative to all other occupations; groups with lower odds are also indicated.

| SSYK2-code | Odds Ratio | 95% CI low | 95% CI high | p-value | English Description |
| --- | --- | --- | --- | --- | --- |
| 83 | 1.5 | 1.1 | 2.0 | 0.02 | Transport and machine operators |
| 23 | 1.4 | 1.3 | 1.6 | <.0001 | Advanced higher education in education |
| 31 | 1.3 | 1.1 | 1.6 | 0.002 | Technology occupations requiring higher education |
| 34 | 1.3 | 1.1 | 1.6 | 0.001 | Culture, recreation and social work (higher education) |
| 26 | 1.3 | 1.1 | 1.4 | <.0001 | Law, culture and social work (advanced education) |
| 42 | 1.3 | 1.1 | 1.5 | 0.004 | Customer service occupations |
| 41 | 1.2 | 1.1 | 1.3 | 0.0007 | Office assistants and secretaries |
| 53 | 1.2 | 1.1 | 1.3 | <.0001 | Care occupations |
| 33 | 1.1 | 1.1 | 1.3 | 0.002 | Economics/admin occupations (higher education) |
| 22 | 1.1 | 1.1 | 1.3 | 0.002 | Advanced higher education in healthcare |
| 24 | 1.1 | 1.0 | 1.2 | 0.006 | Advanced education in economics and administration |
| 94 | 0.7 | 0.5 | 0.9 | 0.002 | Fast food, kitchen and restaurant assistants |
| 91 | 0.3 | 0.3 | 0.4 | <.0001 | Cleaning occupations |

# Table S8. Sensitivity analysis expanding the PCC case definition to include women hospitalized with COVID-19 and/or diagnosed with PCC in specialist outpatient or hospital settings; multivariable ORs with 95% CIs.

| Variable | Odds ratio (95% CI) |
| --- | --- |
| Age | 1.05 (1.04–1.06) |
| Region of birth (ref: Sweden) |  |
| Europe (without Sweden) | 1.17 (0.99–1.38) |
| Africa | 0.55 (0.40–0.76) |
| Asia | 0.84 (0.72–1.01) |
| Americas, Oceania | 1.05 (0.74–1.47) |
| BMI (ref: normal) |  |
| Underweight | 0.99 (0.69–1.42) |
| Overweight | 0.99 (0.87–1.12) |
| Obese (class I) | 0.98 (0.81–1.19) |
| Obese (class II–III) | 1.12 (0.86–1.46) |
| Education (ref: low) |  |
| Medium | 1.14 (0.92–1.43) |
| High | 1.13 (0.90–1.42) |
| Number of visits (ref: 0) |  |
| 1–2 | 1.81 (1.54–2.13) |
| 3–4 | 2.76 (2.31–3.3) |
| 5–6 | 3.94 (3.23–4.81) |
| 7–8 | 4.29 (3.37–5.46) |
| >8 | 5.82 (4.8–7.04) |

# Table S9. Sensitivity analysis excluding individuals with an ED diagnosis recorded during 2014–2020; multivariable ORs with 95% CIs for PCC.

| Variable | Odds ratio (95% CI) |
| --- | --- |
| Age | 1.05 (1.04–1.06) |
| Region of birth (ref: Sweden) |  |
| Europe (without Sweden) | 1.28 (1.07–1.53) |
| Africa | 0.56 (0.39–0.81) |
| Asia | 0.90 (0.74–1.08) |
| Americas, Oceania | 1.21 (0.85–1.73) |
| BMI (ref: normal) |  |
| Underweight | 0.93 (0.61–1.42) |
| Overweight | 1.02 (0.88–1.17) |
| Obese (class I) | 1.01 (0.82–1.25) |
| Obese (class II–III) | 1.18 (0.88–1.58) |
| Education (ref: low) |  |
| Medium | 1.18 (0.92–1.51) |
| High | 1.18 (0.92–1.53) |
| Number of visits (ref: 0) |  |
| 1–2 visits | 1.74 (1.46–2.07) |
| 3–4 visits | 2.76 (2.28–3.34) |
| 5–6 visits | 3.68 (2.96–4.58) |
| 7–8 visits | 4.05 (3.09–5.32) |
| >8 visits | 5.22 (4.14–6.58) |

# Table S10. Sensitivity analysis removing all psychiatric symptom F-codes from the exposure list; adjusted ORs (95% CIs) for PCC, PVFS (G93.3), and ED across covariates and symptom-visit categories.

| Variable | Odds ratio PCC (95% CI) | Odds ratio PVFS (95% CI) | Odds ratio ED (95% CI) |
| --- | --- | --- | --- |
| Age | 1.05 (1.04–1.07) | 1.06 (1.04–1.07) | 1.04 (1.03–1.04) |
| Region of birth (ref: Sweden) |  |  |  |
| Europe (without Sweden) | 0.83 (0.64–1.08) | 1.06 (0.86–1.30) | 0.59 (0.54–0.64) |
| Africa | 0.36 (0.21–0.61) | 0.49 (0.33–0.73) | 0.17 (0.13–0.21) |
| Asia | 0.78 (0.62–0.99) | 0.76 (0.62–0.93) | 0.36 (0.33–0.40) |
| Americas, Oceania | 1.17 (0.75–1.81) | 1.01 (0.67–1.53) | 0.68 (0.57–0.81) |
| BMI (ref: normal) |  |  |  |
| Underweight | 1.08 (0.67–1.76) | 1.08 (0.70–1.66) | 1.18 (1.01–1.37) |
| Overweight | 0.93 (0.77–1.11) | 1.03 (0.88–1.20) | 1.05 (0.99–1.11) |
| Obese (class I) | 0.96 (0.74–1.25) | 0.95 (0.75–1.20) | 1.19 (1.09–1.29) |
| Obese (class II–III) | 1.10 (0.76–1.59) | 1.18 (0.86–1.61) | 1.13 (1.00–1.28) |
| Education (ref: low) |  |  |  |
| Medium | 1.02 (0.75–1.39) | 1.07 (0.82–1.39) | 1.29 (1.15–1.44) |
| High | 1.10 (0.81–1.51) | 1.09 (0.83–1.42) | 1.19 (1.06–1.33) |
| Number of visits (ref: 0) |  |  |  |
| 1–2 | 1.8 (1.5–2.2) | 2.0 (1.6–2.4) | 1.6 (1.5–1.7) |
| 3–4 | 2.6 (2.0–3.3) | 2.8 (2.3–3.5) | 2.5 (2.3–2.7) |
| 5–6 | 4.1 (3.1–5.5) | 4.7 (3.7–6.0) | 3.1 (2.9–3.5) |
| 7–8 | 5.5 (3.9–7.9) | 4.6 (3.3–6.4) | 3.3 (2.9–3.8) |
| >8 | 9.0 (6.7–12.2) | 8.0 (6.0–10.5) | 4.1 (3.6–4.6) |

# Table S11. Sensitivity analysis for PCC additionally controlling for the number of non-symptom primary-care physician visits in 2014–2019; ORs (95% CIs) by symptom-based visit category (exposure) versus 0 visits.

| Variable | Odds ratio (95% CI) |
| --- | --- |
| Number of visits (ref: 0) |  |
| 1–2 visits | 1.72 (1.47­-2.03) |
| 3–4 visits | 2.49 (2.08-2.98) |
| 5–6 visits | 3.40 (2.78-4.16) |
| 7–8 visits | 3.56 (2.78-4.55) |
| >8 visits | 4.41 (3.58-5.42) |

# Table S12. Negative-control outcome analyses: hypothyroidism (ICD-10 E03.9), migraine (ICD-10 G43) and IBS (ICD-10 K53) diagnosed during 2020–2024. Shows ORs (95% CIs) by symptom-visit category, with and without additional adjustment for non-symptom physician visits to assess residual confounding by healthcare utilization.

|  | Hypothyroidism | | Migraine | | | IBS | |
| --- | --- | --- | --- | --- | --- | --- | --- |
|  | Odds ratio (95% CI) | Odds ratio (95% CI), model adjusted for non-symptom physician visits | Odds ratio (95% CI) | Odds ratio (95% CI), model adjusted for non-symptom physician visits | | Odds ratio (95% CI) | Odds ratio (95% CI), model adjusted for non-symptom physician visits |
| Number of visits (ref: 0) |  |  |  | |  |  |  |
| 1–2 | 1.14 (1.06–1.23) | 1.08 (0.99-1.17) | 1.45 (1.35-1.55) | 1.37 (1.28-1.47) | | 1.71 (1.51-1.94) | 1.62 (1.42-1.84) |
| 3–4 | 1.35 (1.23–1.49) | 1.21 (1.09-1.34) | 1.91 (1.76-2.07) | 1.71 (1.57-1.86) | | 2.81 (2.44-3.23) | 2.49 (2.16-2.87) |
| 5–6 | 1.37 (1.20–1.55) | 1.08 (0.94-1.24) | 2.27 (2.05-2.51) | 1.93 (1.74-2.15) | | 3.47 (2.94-4.08) | 2.91 (2.46-3.44) |
| 7–8 | 1.41 (1.20–1.67) | 1.10 (0.92-1.31) | 2.90 (2.57-3.28) | 2.38 (2.10-2.69) | | 4.66 (3.86-5.63) | 3.76 (3.10-4.57) |
| >8 | 1.50 (1.31–1.71) | 1.00 (0.86-1.18) | 3.34 (3.03-3.69) | 2.49 (2.23-2.78) | | 5.10 (4.34-5.98) | 3.68 (3.09-4.39) |

|  | Item No | Recommendation | Page  No |
| --- | --- | --- | --- |
| **Title and abstract** | 1 | (*a*) Indicate the study’s design with a commonly used term in the title or the abstract | 1 |
|  |  | (*b*) Provide in the abstract an informative and balanced summary of what was done and what was found | 1 |
| Introduction | | | |
| Background/rationale | 2 | Explain the scientific background and rationale for the investigation being reported | 2 |
| Objectives | 3 | State specific objectives, including any prespecified hypotheses | 3 |
| Methods | | | |
| Study design | 4 | Present key elements of study design early in the paper | 4 |
| Setting | 5 | Describe the setting, locations, and relevant dates, including periods of recruitment, exposure, follow-up, and data collection | 4 |
| Participants | 6 | (*a*) *Cohort study*—Give the eligibility criteria, and the sources and methods of selection of participants. Describe methods of follow-up  *Case-control study*—Give the eligibility criteria, and the sources and methods of case ascertainment and control selection. Give the rationale for the choice of cases and controls  *Cross-sectional study*—Give the eligibility criteria, and the sources and methods of selection of participants | 4 |
|  |  | (*b*) *Cohort study*—For matched studies, give matching criteria and number of exposed and unexposed  *Case-control study*—For matched studies, give matching criteria and the number of controls per case |  |
| Variables | 7 | Clearly define all outcomes, exposures, predictors, potential confounders, and effect modifiers. Give diagnostic criteria, if applicable | 4-5 |
| Data sources/ measurement | 8* | For each variable of interest, give sources of data and details of methods of assessment (measurement). Describe comparability of assessment methods if there is more than one group | *4* |
| Bias | 9 | Describe any efforts to address potential sources of bias | 5 |
| Study size | 10 | Explain how the study size was arrived at | 6 |
| Quantitative variables | 11 | Explain how quantitative variables were handled in the analyses. If applicable, describe which groupings were chosen and why | 5 |
| Statistical methods | 12 | (*a*) Describe all statistical methods, including those used to control for confounding | 5 |
|  |  | (*b*) Describe any methods used to examine subgroups and interactions | 5 |
|  |  | (*c*) Explain how missing data were addressed | 5 |
|  |  | (*d*) *Cohort study*—If applicable, explain how loss to follow-up was addressed  *Case-control study*—If applicable, explain how matching of cases and controls was addressed  *Cross-sectional study*—If applicable, describe analytical methods taking account of sampling strategy |  |
|  |  | (*e*) Describe any sensitivity analyses | 5 |
